# Status quo of interprofessional education for midwifery and medical students in Germany, Austria, and Switzerland

**DOI:** 10.3205/zma001686

**Published:** 2024-06-17

**Authors:** Merle Linnea Juschka, Caroline Johanna Agricola, Felix Alexander Neumann, Sonja Mohr, Birgit-Christiane Zyriax

**Affiliations:** 1 University Medical Center Hamburg-Eppendorf, Midwifery Science-Health Care Research and Prevention, Institute for Health Service Research in Dermatology and Nursing, Hamburg, Germany; 2 University Medical Center Hamburg-Eppendorf, Faculty of Medicine, Dean's Office for Student Affairs, Hamburg, Germany

**Keywords:** IPE, curriculum development, midwifery, medicine, Germany, Austria, Switzerland

## Abstract

**Objectives:**

The care of women and their families around childbirth requires effective interprofessional collaboration of the midwifery and medical profession. Given the academisation of midwifery, early interaction between students of midwifery and medicine is both necessary and feasible. As there is a lack of comprehensive data on interprofessional education (IPE) for midwifery and medical students at higher education institutions in Germany, Austria, and Switzerland (DACH region), the aim was to identify existing IPE activities, and their curricular determination.

**Methods:**

The exploratory study was conducted in the DACH region over three months (Dec. 2022-Feb. 2023). Higher education institutions offering midwifery science and/or medicine were invited to participate in a web-based survey. The questionnaire focused on the structure and curricular implementation of IPE courses, on cooperation, financial support and more.

**Results:**

A total of 58 out of 96 invited institutions (60%) participated in the survey, of which 34 (59%) offered IPE. Eighteen institutions (19%) offered 32 IPE courses for midwifery and medical students through cooperation within faculty (*n*=8) and between faculties (*n*=10). Notably, most of these IPE courses (60%) were integrated into the required curriculum of both study programmes. Most IPE courses were offered without financial support (71%).

**Conclusion:**

The current status quo highlighted the existence of numerous IPE offers for midwifery and medical students in the DACH region that warrant further curricular integration of proven and well-established best practice examples to further enhance these initiatives.

## Zusammenfassung

**Ziele:**

Die Versorgung von Frauen und ihren Familien rund um die Geburt erfordert eine intensive Zusammenarbeit von Hebammen und Ärzt*innen. Die Akademisierung des Hebammenberufs hat die Möglichkeit geschaffen, dass Studierende der Hebammenwissenschaft und der Medizin bereits während des Studiums in Kontakt kommen können. Da es bisher für Deutschland, Österreich und die Schweiz (DACH-Region) keine umfassende Datenlage zum Stand interprofessionellen Lernens (IPL) für Medizin- und Hebammenwissenschaftsstudierende gibt, war das Ziel der vorliegenden Studie, die Erfassung ebendieser Aktivitäten und deren curricularer Verankerung.

**Methoden:**

Die explorative Studie wurde in der DACH-Region über einen Zeitraum von drei Monaten (Dez. 2022-Feb. 2023) durchgeführt. Hochschulen, die die Studiengänge Hebammenwissenschaft und/oder Medizin anbieten, wurden eingeladen an der Online-Befragung teilzunehmen. Der Fragebogen fokussierte die Struktur, curriculare Verankerung, zugrundeliegende Kooperationen, Finanzierung und weitere Aspekte der interprofessionellen Lehr-Lern-Angebote.

**Ergebnisse:**

Insgesamt beteiligten sich 58 von 96 eingeladenen Hochschulen (60%) an der Umfrage, von denen 34 (59%) angaben, IPL anzubieten. Auf 18 dieser Hochschulen (19%) verteilten sich 32 interprofessionelle Kursangebote, die für Hebammenwissenschafts- und Medizinstudierende gestaltet waren, entweder durch Kooperationen innerhalb der Fakultät (*n*=8) oder zwischen Fakultäten (*n*=10). Der Großteil der Angebote war in beiden Studiengängen im Pflichtcurriculum verankert (60%). Die meisten Kurse wurden nicht finanziell unterstützt (71%).

**Schlussfolgerung:**

Die Bestandsaufnahme verdeutlicht eine Vielzahl von interprofessionellen Lehr-Lernangeboten für Hebammenwissenschafts- und Medizinstudierende in der DACH-Region. Die Betrachtung von Best-Practice-Beispielen kann dabei helfen, die Initiativen in der gesamten Region weiterzuentwickeln und flächendeckend curricular zu verankern.

## Background

Providing comprehensive care for women and families around childbirth requires intensive collaboration between the midwifery and medical profession ([1], p.53-54). In practice, this becomes difficult when individuals lack a deep understanding of each other’s roles and professional cultures, potentially leading to negative stereotypes rather than mutual respect and understanding [2]. Interprofessional education (IPE) addresses this difficulty at the academic level by preparing students from different professions to “learn about, from, and with each other” [3]. Successful IPE initiatives have already been conducted in the field of obstetrics [4], [5], associated with improved patient safety, outcomes, patient and job satisfaction [6], [7], [8], [9]. Thus, promoting IPE for midwifery and medical students is an important factor to achieve the German National Health Goal 2017 “health around childbirth” ([1], p.53-54), [10]. The relevance of IPE for both professions is emphasized by its inclusion in the National Competence Based Learning Objectives Catalogue Medicine (NKLM) 2.0 [https://nklm.de/zend/menu] and Study and Examination Regulations for Midwives (HebStPrV) [https://www.gesetze-im-internet.de/hebg_2020/BJNR175910019.html]. In Austria, the midwifery profession has already been academised in 2006 with the enactment of the adjusted Austrian Midwifery Law [https://www.ris.bka.gv.at/GeltendeFassung.wxe?Abfrage=Bundesnormen&Gesetzesnummer=10010804]. In German-speaking Switzerland, midwives were incorporated into higher education institutions in 2008 under the Healthcare Professions Act [https://www.fedlex.admin.ch/eli/cc/2020/16/de] and Ordinance on the Higher Education Act [https://www.fedlex.admin.ch/eli/cc/2014/691/de]. At last, the recent academisation of midwifery in Germany, brought about by the reformed Midwifery Act (HebG) in 2019 [https://www.gesetze-im-internet.de/hebg_2020/BJNR175910019.html] may facilitate collaboration between study programmes. However, unlike other countries, higher education for healthcare professions in Germany remains predominantly mono-professional and IPE has yet to be integrated into the curricula [8], [11], [12]. Several factors obstruct IPE implementation, including the heterogeneity of study groups in terms of cohort sizes per semester, different duration of the programs (midwifery science 6-8 semesters [13] vs. medicine 12.5 semesters [https://www.gesetze-im-internet.de/_appro_2002/BJNR240500002.html]), and differing curricula, such as the large amount of clinical placements in midwifery education [14]. Because of these barriers, faculties need to be convinced of the importance of IPE as they need qualified instructors, additional time, room, and tools, but are often constrained by limited financial and personnel resources. Consequently, effective approaches must be developed to connect different professions, necessitating a reconfiguration of curricula originally designed for mono-professional education [15].

The DACH region, comprising Germany, Austria, and Switzerland, has comparable healthcare and educational systems, as well as a similar intention to catch up with international efforts in implementing IPE. However, the region faces a lack of institutionalisation, networking, research on IPE, and curricular reforms [16]. Several IPE initiatives exist in the DACH region as analysed in the GMA Committee’s Position Paper of 2022 [17], and IPE was facilitated through the support of the Robert Bosch foundation [18]. Nevertheless, there is a notable lack of activities involving the midwifery profession. 

Key characteristics of IPE activities have recently been reported in a review [15]. They are typically conducted through co- or team-teaching, predominantly delivered face-to-face, favouring interactive over didactic formats, or sometimes combining both. While information on the implementation is limited, most IPE activities were integrated into the curricula. Some are mandatory for specific groups of students while being optional for others. Bogossian et al. [15] emphasized that focusing on interprofessional socialisation is as important as practical topics. Another international review identified four best practice examples for undergraduate training, particularly for midwifery and medical students [19]. Single initiatives for IPE between midwifery and medical students were reported, including clinical training, childbirth simulations, case studies, or web-based training [4], [5], [20], [21], [22], [23], [24], [25], [26], [27]. Nonetheless, there is no comprehensive study providing an overview of all IPE activities for midwifery and medical students. Furthermore, transferring international IPE initiatives to the DACH region may not be feasible due to differences in healthcare system structures and educational systems. Hence, the aim of this survey was to evaluate the status quo of IPE for midwifery and medical students and the current state of its curricular implementation in the DACH region.

## Methods

### Setting and participants

German-speaking universities and universities of applied sciences in the DACH region that offer either one or both study programmes were included in this study. Through online research following a four-eyes principle, a comprehensive list of higher education institutions (N=96) that met these inclusion criteria were compiled. Subsequently, these institutions were invited to voluntarily participate in an online questionnaire between December 2022 and March 2023.

### Processes, variables, and analyses

Contact details were obtained from the institution’s faculty websites, with a primary focus on contacting faculty members with expertise in IPE or those involved in teaching coordination. In cases in which these persons could not be identified, study programme directors or relevant members of the deanery were contacted. Given the absence of a validated questionnaire on this topic, a self-developed questionnaire was created, consisting of 27 items. The questionnaire was pre-tested by members of the Midwifery committee of the Society of Medical Education (GMA). The detailed questionnaire covered a wide area of topics, including curricular implementation, cooperation, financial support, additional staff and training for IPE, planned IPE activities, and the structure of courses. This included items like participating professions, course format (lecture, seminar etc.), IP facilitators (“Who is responsible for conducting the specified teaching event?”), contextual focus (knowledge transfer, practical skill training, attitude formation), and more. Participants either participated anonymously or voluntarily disclosed their location. An open-ended question was integrated to provide recommendations for future needs and wishes. The quotes were categorized inductively into three themes: faculty development, IPE content, and collaboration. The quotes in the first theme were further sorted based on the four different types of faculty development from Centra [28] and summarized by Amundsen et al. [29]: “personal (interpersonal skills, career development, and life planning issues); instructional (course design and development, instructional technology); organizational (ways to improve the institutional environment to better support teaching); and professional (ways to support faculty members so that they fulfil their multiple roles of teaching, research, and service)”. The quantitative data analyses were limited to descriptive statistics due to the small sample size. No systematic patterns of missing or implausible data were identified in the sample. Whenever missing and implausible data occurred in a variable, these cases were not accounted for in the respective analysis. The statistical analysis was performed using IBM SPSS^®^ (version 29.0.1.0).

## Results

### Sample

**Figure 1:**
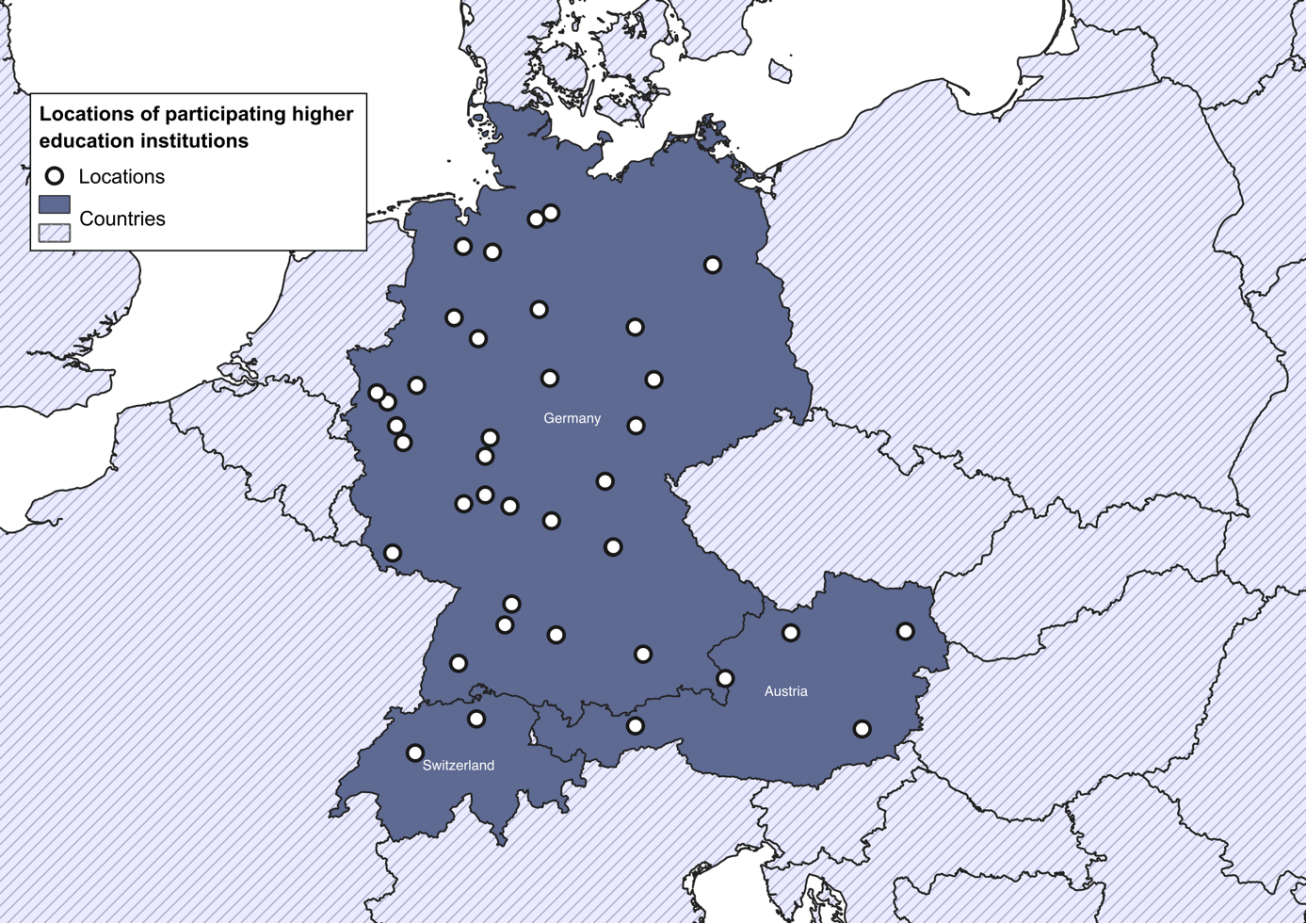
Locations of participating universities and universities of applied sciences in Germany, Austria and Switzerland (created by Marie Sander) Note: 96 higher education institutions were invited to participate in this study. 58 institutions (return rate: 60%) from Germany (n=37), Austria (n=8) and Switzerland (n=3) participated in the survey. Of the participating institutions, ten did not disclose their location.

In total, 58 of the 96 invited universities and universities of applied sciences (response rate 60.4%) were represented in the survey. The participants’ institutions were located in Germany, Austria and Switzerland (see table 1 (Tab. 1) and figure 1 (Fig. 1)). Most of the participating institutions (83%, n=48) disclosed their location. The distribution across the three countries is presented in figure 1 (Fig. 1). The survey was completed by teaching and research staff (*n*=41) or by members of the administration/deanery (*n*=14). 

### Reported courses

**Figure 2:**
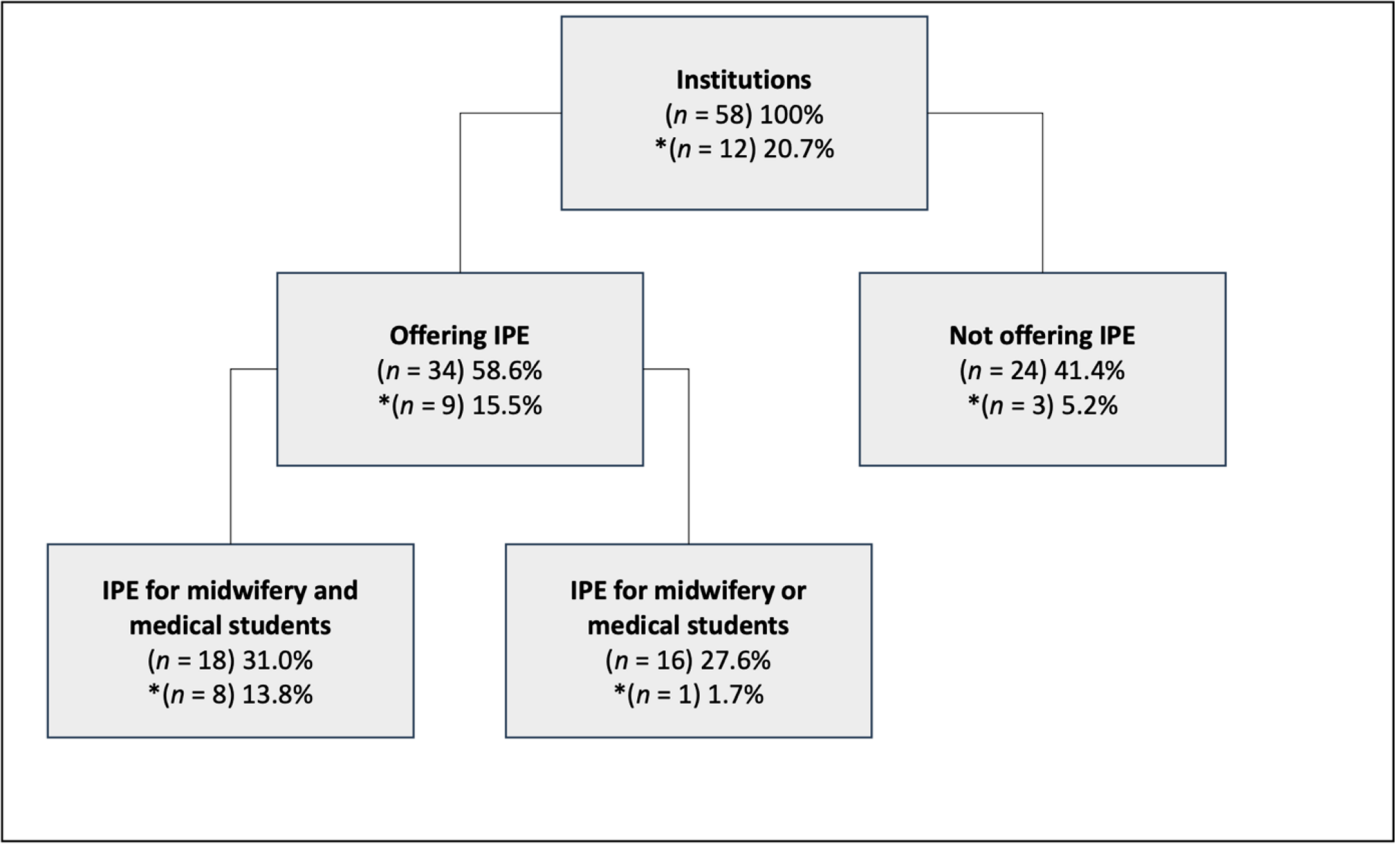
Overview of Interprofessional Education (IPE) offered by participating institutions Note: Percentage figures in the entire graph refer to the total size of N=58 participating institutions. Since there are institutions that have offered IPE for both professions together as well as separately, the number of institutions offering IPE in different constellations should not be understood as summands. *Institutions with both study programmes midwifery science and medicine.

Altogether, 34 institutions offered IPE with a total of 58 different interprofessional courses which included midwifery and/or medical students. In the analysis, the term “course” encompasses the range of IPE offerings, which vary greatly in length. In 32 IPE courses both professions study together, of which 21 were exclusively designed for midwifery and medical students. Most of the reported courses are currently taking place (67%, *n*=21), while approximately 16% (*n*=5) are at the planning stage, and very few were conducted in the past. Fifteen institutions offered more than one IPE activity. Additionally, 26 IPE courses were documented that either involved midwifery (*n*=14) or medical students (*n*=12) in combination with other healthcare professions. The analysis in this section focuses on the identified 32 IPE courses for midwifery and medical students, in some cases with additional professions, that were provided by eighteen institutions (*n*=6 universities of applied sciences; *n*=12 universities) (see figure 2 (Fig. 2) and table 1 (Tab. 1), table 2 (Tab. 2) and table 3 (Tab. 3)). 

There was a difference regarding the point in time at which the courses were designed in midwifery and medical programmes. In midwifery programmes, 90% (*n*=28) of courses were designed for students in a particular semester, predominantly the first (28%, *n*=9), second (13%, *n*=4), third (19%, *n*=6) or fourth (13%, *n*=4) semester. In medical education, 14 courses included students from various semesters. The remaining 17 courses (N/A=1) were open to students from certain semesters throughout medical education. On average, far more medical than midwifery students participated in the IPE courses (see table 2 (Tab. 2)). Most courses were led by peer- or interprofessional co-teachers (see table 3 (Tab. 3)). 

The participants reported many different course formats, mainly skills-lab-units or a mixture of different formats (see table 3 (Tab. 3)). Most courses (*n*=23) could be completed without an examination. In three cases only one of the participating professions was supposed to take an exam.

The main mode of delivery was face-to-face. The number of teaching units (one unit equals 45 minutes) varied largely in the sample (mode=2 units; see table 3 (Tab. 3)).

Participants were asked about the focus of their courses. Two categories occurred most in this multiple choice question: practical skill training (68.8%, *n*=22) and attitude formation (71.9%, *n*=23). In 18.8% (*n*=6) of courses, the focus was knowledge transfer. Furthermore, most courses were part of the required curriculum (60%, *n*=18) in both study programmes. 

Lastly, there was a difference regarding the kind of cooperation the courses were based on: either collaboration within faculties or collaboration between faculties. 

### Collaboration within faculties 

Nine of the twelve participating institutions that offer both study programmes provided IPE (see figure 2 (Fig. 2)). Eight of them offered eleven courses for both midwifery and medical students. Additionally, nursing students/apprentices participated in four and digital health management students in one of these courses. Most of the courses were launched between 2021 and 2023. Four reported courses are about to start in 2023.

### Collaboration between faculties

The remaining ten institutions, providing either midwifery science or medicine, offered 21 IPE courses for midwifery and medical students in cooperation with another institution. Thirteen of these courses were exclusively designed for midwifery and medical students. Nursing students/apprentices participated in eight courses. Some courses also integrated the professions nutritional science, physiotherapy, logopaedics, and radio technology assistants. Some faculties began collaborating before the structure of midwifery education was reformed in 2019, starting in 2014. Most courses were launched in 2017 (42%, *n*=8), while two are about to start in 2023 and 2025.

### Organizational factors

Eight of all participating institutions (14%) have created jobs for the implementation and coordination of IPE. 24% (*n*=14) of the institutions conducted workshops for IP facilitators. Most institutions with IPE offers did not receive financial support for their IPE courses (71%, *n*=24). The others either received unlimited, limited internal or limited external financial support.

### Planned projects

Plans for future IPE were assessed in an open question. 30 institutions are currently planning new or additional IPE activities. These include workshops on interprofessional interaction in the delivery room and/or maternity ward, interprofessional training wards, case studies, and the introduction of a joint bachelor thesis in interprofessional teams. In total, six universities of applied sciences declared no intentions to implement IPE at their institution. Three participants stated that they are not opposed to the implementation of IPE, but face too many barriers. For example, one participant wrote that a collaboration would probably fail because the medical faculty lacks initiative: “Cooperation with the medical faculty is very difficult. Despite repeated initiatives, the medical faculty does not seem to be willing to cooperate at all”. Another participant wrote that the “basic structural framework conditions still need to be clarified” for the implementation of IPE.

### Wishes

Topics from the areas of Faculty Development (*n*=47 codings), IPE Content (*n*=5 codings), and Collaboration (*n*=6 codings) were mentioned by 30 institutions when asked about wishes regarding IPE. Wishes regarding Faculty Development are presented in table 4 (Tab. 4). Participants also wished for best practice examples and IPE concepts that would help in the instructional design of IPE. One participant mentioned “The main problem is [...] to identify topics that are interesting for both professions at their individual levels of training.”. Lastly, the collaboration category comprised wishes for the facilitation of collaboration between institutions in the form of financial resources, commitment, and readiness of faculty members. One participant expressed “Other [...] study programmes should [...] ideally devote similar resources to IPE.”

## Discussion

The aim of this study was to evaluate the status quo of IPE for midwifery and medical students in the DACH region and to assess to what extent IPE has already been implemented in the curriculum. The survey provides an overview of 58 out of 96 higher education institutions that provide midwifery science and/or medicine in Germany, Austria, and German-speaking parts of Switzerland. 58.6% (*n*=34) of the participating institutions currently offer IPE with only a third receiving financial support. For guaranteed long-term implementation, all projects currently in the planning phase should receive the necessary support, as demonstrated by past fundings [18]. In sum, 21 out of 57 medical faculties in the DACH region offer midwifery science programmes (36.8%). Twelve (57.1%) participated in the survey of which eight provided IPE for midwifery and medical students. An additional number of ten institutions provided IPE through collaboration between faculties. Results showed that four universities did not offer IPE or reported any planned projects for midwifery and medical students although they offer both study programmes. Collaboration within faculty may facilitate IPE for midwifery and medical students, addressing the challenges related to the heterogeneity of programmes at different institutions (e.g. universities versus universities of applied sciences), which may be a compelling reason for the integration of midwifery science at medical faculties [30], [31]. In this study, 21 courses were identified that were implemented through cooperation between faculties. The limited availability of IPE offerings for midwifery students might be linked to the evident challenges associated with collaboration between faculties. As highlighted in the GMA Committee’s Position Paper of 2022 [17], the majority of IPE tends to target professions like medicine, nursing, and physiotherapy, often neglecting other crucial healthcare professions like midwifery. Nevertheless, this survey identified 32 courses designed for midwifery students, with 35% of these involving professions beyond medicine. The current status quo unveiled several key findings, as summarized in table 5 (Tab. 5).

### Timing and focus of IPE

There is no consensus on the optimal timing for IPE. Gilbert [32] argued that IPE might be rather counterproductive in the early stages, as students need to first grasp the fundamentals of their own profession. In contrast, Charles et al. [33] and Park et al. [34] proposed the introduction of IPE at an early stage to foster positive student attitudes as well as to expose students to other professions in courses where full interaction or understanding is not necessary. Charles et al. [33] also suggested that IPE should evolve throughout the programmes when both professions are more experienced, and ready for attitude formation in a clinical setting. 

In the present study, many institutions matched midwifery students from the first to the fourth semester (73%, *n*=23) with medical students from various semesters (50%, *n*=14). This might be attributed to the heterogeneous programme structures and length leading to varying levels of training, prior knowledge, and clinical experience. Having more heterogeneous student groups from various semesters may not only bridge these differences but also better prepare them for interprofessional collaboration in practice [35]. However, in line with the differing cohort sizes, more medical than midwifery students were involved. Therefore, the handling of different participant numbers remains a challenge [14] and best practice examples are required to address this issue. The participation of midwives and other healthcare professions from numerous semesters could offer a solution. As shown, a longitudinal approach of IPE might be particularly beneficial for students. This was observed in only five cases in the present study. Many of the reported courses focus on attitude formation in the early stages of programmes. Practical skill trainings were the second most reported approach. Bogossian et al. [15] emphasized that both should be integrated, and that practical skill trainings might be more effective with experienced students. In contrast, topics that no profession has prior knowledge of could be introduced at an early stage. 60% of courses were part of the required curriculum of both professions. Previously reported numbers in the German context were considerably lower [18]. However, most of the reported IPE courses are single activities with a small number of teaching units, prompting the question of whether already established scheduled courses can be converted into IPE courses, as well as highlighting the need for a more substantial shift in the curriculum towards sustainable IPE integration [36]. These findings align with the recommendation of VanKuiken et al., who advocated finding innovative strategies for integrating IPE into the curriculum, making it a mandatory rather than optional addition [37]. Furthermore, the Action Plan Implementation of the IMPP also suggests an interprofessional master plan at medical faculties, with a total of 50 teaching units focusing on interprofessional cooperation and communication [32].

### IP facilitators

A significant number of courses in the present study were led by peer teachers (*n*=11), which contrasts with findings of existing IPE reviews [15]. Peer teaching offers the advantage of learning not only “with each other” but also “from each other”, which aligns with the three key elements of IPE [3]. It facilitates interprofessional confrontation and collaboration [38] and has been proven to be beneficial for both students and peer teachers [39]. The potential of this approach in the context of IPE implementation should be further examined in future studies. Additionally, it is important to mention that 16 IPE courses were taught mono-professionally, even though the involvement of “educators [...] from 2 or more health professions” is recommended [35]. The survey’s findings suggest that the financial resources required to employ 2 teachers must be ensured and the advantages of co-teaching must be better promoted. For example, interprofessional co-teachers can more easily identify topics relevant to both professions [40], are able to foster a sense of equality and promote a positive working relationship [41]. Therefore, they can act as role models for interprofessional collaboration.

### Course format and examinations

There was a predominance of interactive course formats in the present study that has already been reported in previous research [15]. Although, examinations are described as conducive to learning and as goal-oriented [42], very few courses included examinations in this study. This may be provoked by the focus on interprofessional socialisation rather than knowledge transfer. 

### Faculty development

Institutions must allocate financial resources to ensure IPE implementation with qualified staff [42]. Only a few institutions have created jobs specifically for IPE implementation, despite the need for additional staff to prevent an excessive workload and to ensure effective IPE implementation [43]. Furthermore, only 24% (*n*=14) have conducted IPE workshops, even though a mandatory IPE training for IP facilitators, including peer teachers, is recommended [42]. Results of the qualitative analyses substantiate, that faculty development, IPE structure, and collaboration are seen as most relevant for future endeavours regarding the implementation of IPE. These findings support the results from previous studies [14], [15]. 

### Limitations and future research

The survey was conducted using a self-developed questionnaire and did not include inquiries about reasons for non-participation. Future studies could provide first comparable data, and including such questions may increase participant numbers and reach further locations with IPE offerings. Additionally, it was limited to German-speaking higher education institutions in the DACH region. The focus were midwifery and medical students, which represents only a small aspect of the broader need for IPE among various healthcare professions. Future research should consider a more comprehensive, international approach, encompassing a wider range of healthcare professions. Overall, there is still a need for proven and well-established IPE concepts and best practice examples in the DACH region. The present study showed that there are plenty of established courses that are worth looking into.

## Conclusion

The current status quo highlighted the existence of numerous IPE offers for midwifery and medical students in the DACH region that warrant further curricular integration. In order to establish IPE longitudinally within the regular curriculum, mandatory regulations such as the NKLM 3.0 [44], which should be binding as per the Licensing Regulations for Doctors (ÄApprO), are imperative. Additionally, IPE concepts should be shared as best practices for institutions that have yet to implement IPE. 

## Notes

### Shared authorship

Sonja Mohr and Birgit-Christiane Zyriax contributed equally to this work and share last authorship.

### Authors’ ORCIDs

- Merle Linnea Juschka: [0009-0007-5778-3930]
- Caroline Johanna Agricola: [0000-0001-8347-2211]
- Felix Alexander Neumann: [0000-0003-3107-075X]
- Birgit-Christiane Zyriax: [0000-0002-5377-5956]

### Funding

We acknowledge financial support from the Open Access Publication Fund of UKE - Universitätsklinikum Hamburg-Eppendorf.

## Acknowledgements

The authors gratefully acknowledge the participants taking part in this survey to generate an overview of the existing IPE courses for midwifery and medical students in German-speaking countries. The authors extend their gratitude to Marie Sander for her contribution in crafting the map depicting participating institutions in the DACH region (see figure 1 (Fig. 1)). 

## Competing interests

The authors declare that they have no competing interests. 

## Figures and Tables

**Table 1:**
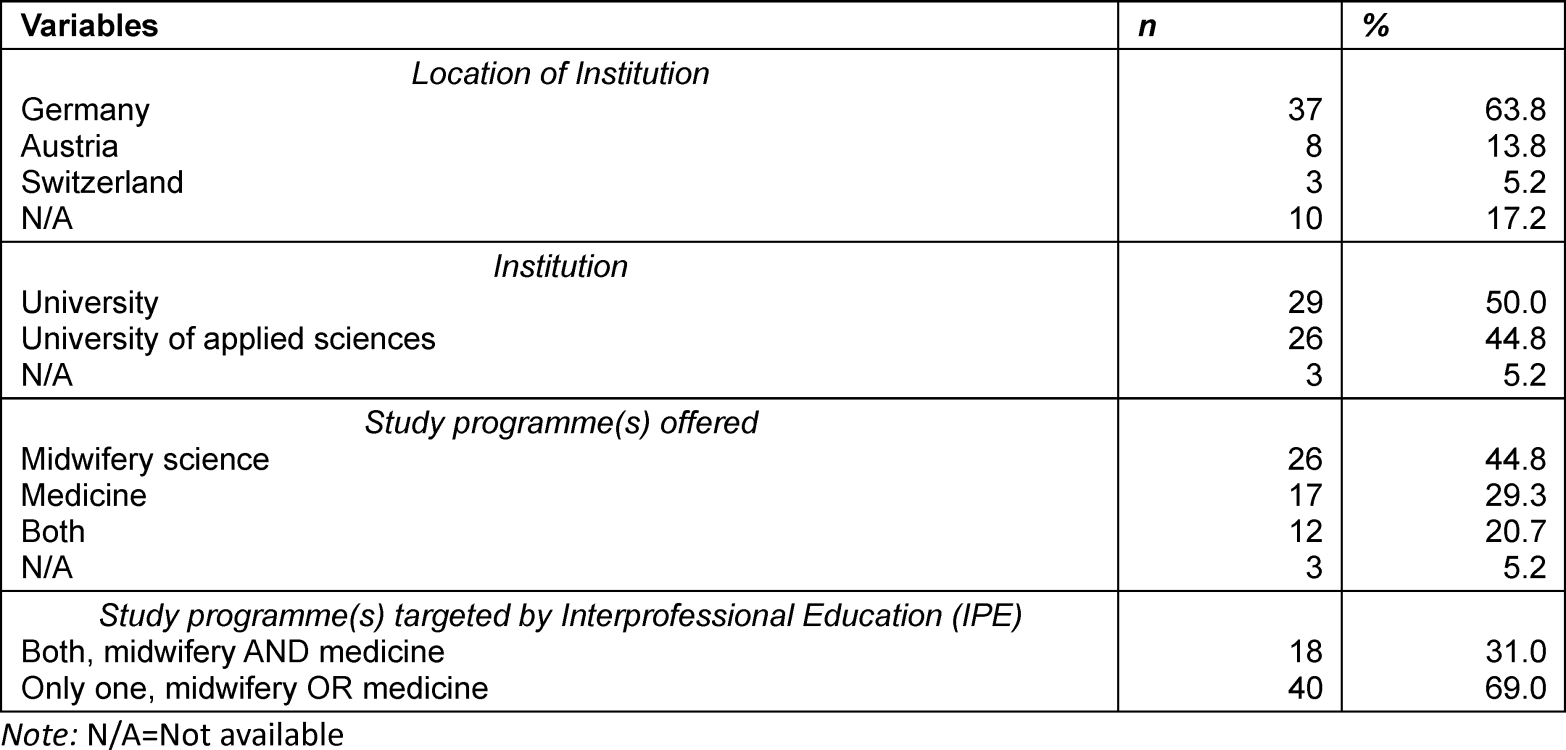
Institution-Related characteristics (N=58)

**Table 2:**
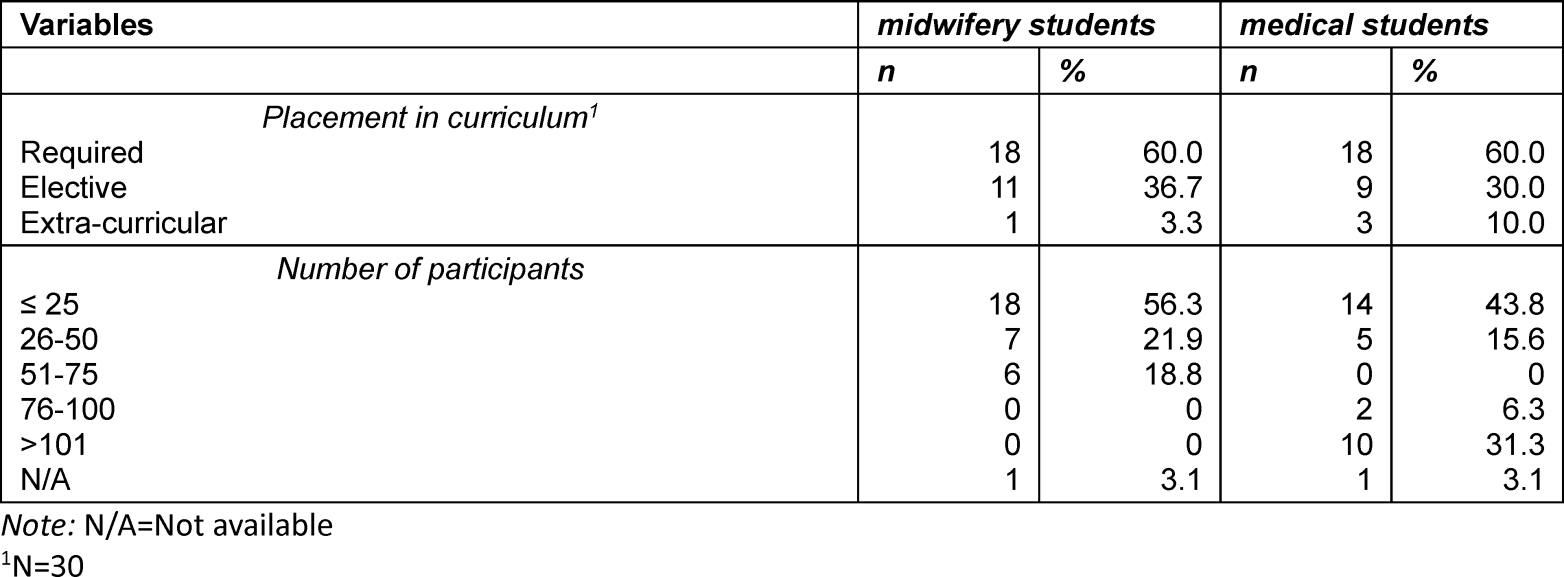
Characteristics of reported Interprofessional Education (IPE) courses 1 (N=32)

**Table 3:**
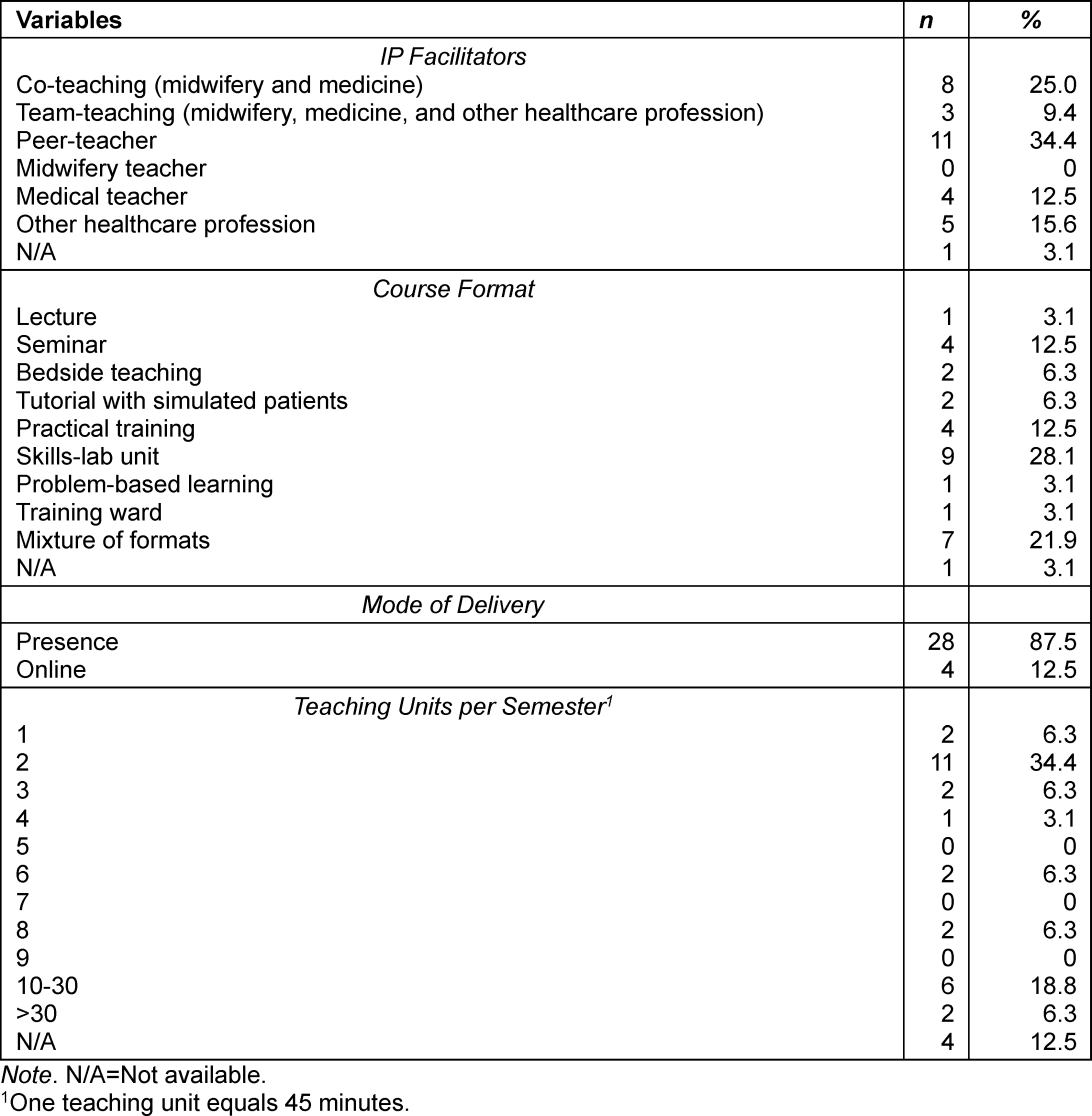
Characteristics of reported Interprofessional Education (IPE) courses 2 (N=32)

**Table 4:**
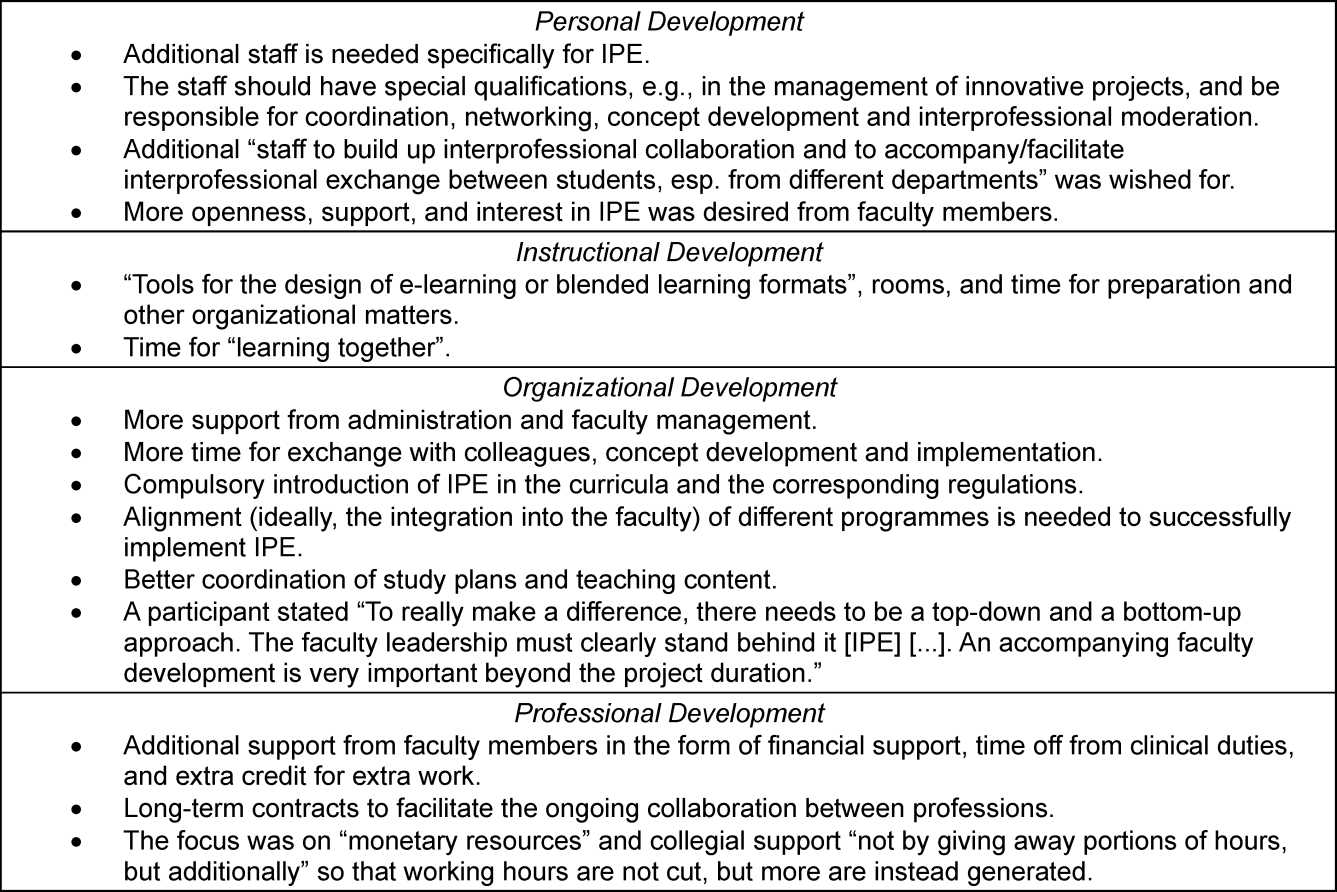
Wishes regarding further implementation of Interprofessional Education (IPE)

**Table 5:**
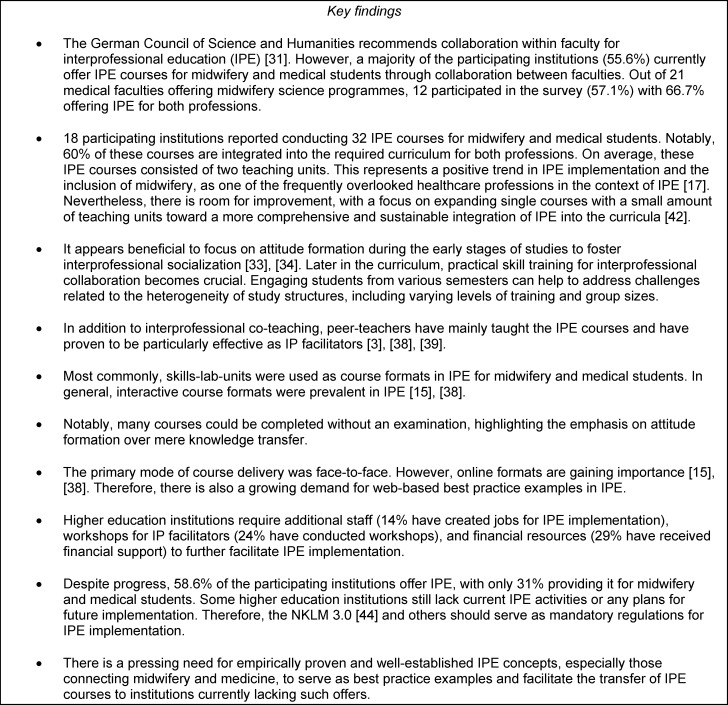
The survey’s key findings
